# Supplementary material for: Effects of plyometric training on technical skill performance among athletes: A systematic review and meta-analysis
Source: PLoS One. 2023 Jul 17;18(7):e0288340. doi: 10.1371/journal.pone.0288340 (PMC10351709; doi:10.1371/journal.pone.0288340)
Supplement: S1 Table — (DOCX) [file pone.0288340.s001.docx]

**Table S1. Detailed search strategy.**

**Searched on 3 May 2023**

| **Databases** | **search strategy** | **Results** |
| --- | --- | --- |
| PubMed | (((("plyometric training"[Title/Abstract] OR "plyometric exercise*"[Title/Abstract] OR "stretch-shortening cycle"[Title/Abstract] OR "jump training"[Title/Abstract])) AND (("athletic performance" [Title/Abstract] OR "technical skill∗" [Title/Abstract] OR "skill∗"[Title/Abstract] OR "technique" [Title/Abstract] OR "performance" [Title/Abstract]))) AND (athlete* [Title/Abstract] OR player* [Title/Abstract])) | 490 |
| Web of Science Core Collection | (((AB=(("plyometric training" OR "plyometric exercise*" OR "stretch-shortening cycle" OR "jump training"))) AND AB=((“athletic performance” OR “technical skill∗” OR “skill∗” OR “technique” OR “performance”))) AND AB=( athlete* OR player*)) | 503 |
| SPORTDicus | AB ( "plyometric training" OR "plyometric exercise*" OR "stretch-shortening cycle" OR "jump training" ) AND AB ( "athletic performance" OR "technical skill∗" OR "skill∗" OR "technique" OR "performance" ) AND AB ( athlete* OR player* ) | 488 |
| Scopus | TITLE-ABS-KEY("plyometric training" OR "plyometric exercise*" OR "stretch-shortening cycle" OR "jump training") AND TITLE-ABS-KEY("athletic performance" OR "technical skill∗" OR "skill∗" OR "technique" OR "performance") AND TITLE-ABS-KEY(athlete* OR player*) | 1020 |
| Total |  | 2501 |
