# Supplementary material for: Effects of plyometric training on technical skill performance among athletes: A systematic review and meta-analysis
Source: PLoS One. 2023 Jul 17;18(7):e0288340. doi: 10.1371/journal.pone.0288340 (PMC10351709; doi:10.1371/journal.pone.0288340)
Supplement: S2 Table — (DOCX) [file pone.0288340.s002.docx]

| **Table S2 The data used for meta-analysis** |  |  |  |  |  |  |  |  |  |  |  |  |
| --- | --- | --- | --- | --- | --- | --- | --- | --- | --- | --- | --- | --- |
| **Study** | **Sport** | **Test** | **EG pre-test** |  | **EG post-test** |  | **n** | **CG pre-test** |  | **CG post-test** |  | **n** |
|  |  |  | Mean | SD | Mean | SD |  | Mean | SD | Mean | SD |  |
| Campo et al. (74) | Soccer | Kicking velocity | 70 | 2.4 | 77.6 | 2.0 | 10 | 75.8 | 1.5 | 73.4 | 0.9 | 10 |
| Sedano (75) | Soccer | Kicking velocity | 97.1 | 3.2 | 102.7 | 4.3 | 11 | 98.3 | 3.7 | 100.1 | 6.1 | 11 |
| Ramı´rez-Campillo et al. (83) unilateral | Soccer | Kicking velocity | 59.2 | 18.4 | 63.3 | 19.14 | 12 | 51.9 | 14.2 | 54.1 | 16.6 | 5 |
| Ramı´rez-Campillo et al. (83) bilateral | Soccer | Kicking velocity | 59.9 | 10.8 | 69.9 | 22.8 | 16 | 51.9 | 14.2 | 54.1 | 16.6 | 5 |
| Ramı´rez-Campillo et al. (83 ) unilateral+ bilateral | Soccer | Kicking velocity | 61.8 | 19.6 | 66.3 | 14.9 | 12 | 51.9 | 14.2 | 54.1 | 16.6 | 4 |
| Ramı´rez-Campillo et al. (84) vertical | Soccer | Kicking velocity | 60.6 | 6.0 | 64.9 | 5.3 | 10 | 63 | 15.5 | 65.9 | 15.5 | 3 |
| Ramı´rez-Campillo et al. (84) horizontal | Soccer | Kicking velocity | 59.8 | 16.1 | 66.4 | 13.1 | 10 | 63 | 15.5 | 65.9 | 15.5 | 3 |
| Ramı´rez-Campillo et al. (84) vertical + horizontal | Soccer | Kicking velocity | 58.3 | 15.9 | 68.9 | 11.7 | 10 | 63 | 15.5 | 65.9 | 15.5 | 4 |
| Ramı´rez-Campillo et al. (85) with progressive volume | Soccer | Kicking velocity | 68.3 | 15.4 | 70.9 | 13.14 | 8 | 64.4 | 13.9 | 63.6 | 19.6 | 4 |
| Ramı´rez-Campillo et al. (85) without progressive volume | Soccer | Kicking velocity | 67.1 | 16.3 | 72.6 | 16.3 | 8 | 64.4 | 13.9 | 63.6 | 19.6 | 4 |
| Ramirez-Campillo et al. (91) one session | Soccer | Kicking velocity | 65.1 | 9.0 | 70.6 | 8.9 | 7 | 67.3 | 7.2 | 68.9 | 7.5 | 3 |
| Ramirez-Campillo et al. (91) two session | Soccer | Kicking velocity | 63 | 9.5 | 68.9 | 11 | 8 | 67.3 | 7.2 | 68.9 | 7.5 | 4 |
| Ramirez-Campillo et al. (95) combined surf | Soccer | Kicking velocity | 59.8 | 18.2 | 67.9 | 20.7 | 8 | 62 | 15.4 | 61.6 | 13.4 | 4 |
| Ramirez-Campillo et al. (95) single surf | Soccer | Kicking velocity | 71.1 | 14.9 | 77.1 | 15.9 | 8 | 62 | 15.4 | 61.6 | 13.4 | 4 |
| Wee et al. (100) | Soccer | Kicking velocity | 17.88 | 1.72 | 21.03 | 2.49 | 10 | 19.21 | 2.1 | 21.23 | 2.18 | 9 |
| Behringer et al. (19) | Tennis | Serving velocity | 127 | 12.5 | 131.2 | 12.3 | 10 | 115.1 | 19.1 | 109.0 | 18 | 10 |
| Carter et al. (72) | Baseball | Throwing velocity | 28.6 | 3.4 | 34 | 2.4 | 12 | 28.3 | 4.6 | 30.7 | 3.2 | 11 |
| Escamilla et al. (76) | Baseball | Throwing velocity | 33 | 2.3 | 33.7 | 5.1 | 14 | 32.6 | 3.1 | 32.5 | 2.5 | 15 |
| Chelly et al. (81) | Handball | Throwing velocity | 28.6 | 3.4 | 34 | 2.4 | 12 | 28.3 | 4.6 | 30.7 | 3.2 | 11 |
| De Villarreal et al. (82) | Water polo | Throwing velocity | 60.2 | 3.6 | 61.1 | 3.4 | 10 | 58.3 | 3.6 | 59.7 | 3.7 | 9 |
| Ramos-Veliz et al. (86) | Water polo | Throwing velocity | 50.11 | 1.04 | 53.55 | 1.11 | 11 | 48.01 | 2.52 | 49.17 | 2.15 | 10 |
| De Villareal et al. (87) | Water polo | Throwing velocity | 51.1 | 6.0 | 57 | 5.0 | 10 | 52 | 5.8 | 61.1 | 5.3 | 10 |
| Aloui et al. (97) | Handball | Throwing Velocity | 21.6 | 2.6 | 26 | 3.1 | 14 | 20.7 | 2.8 | 22 | 2.9 | 15 |
| Alp and Ozdinc (98) | Handball | Throwing Velocity | 73.9 | 3.98 | 79 | 3.36 | 10 | 75.2 | 3.88 | 75.5 | 3.4 | 10 |
| Michailidis et al. (78) | Soccer | Kicking Distance | 23.5 | 7.3 | 28.9 | 8 | 24 | 23.9 | 7.8 | 24.9 | 9.2 | 21 |
| Ramírez-Campillo et al. (79) in-season | Soccer | Kicking Distance | 32.7 | 7.7 | 35.3 | 8.5 | 38 | 30.9 | 7.4 | 30.8 | 6.5 | 38 |
| Ramírez-Campillo et al. (80) 30s interset rest | Soccer | Kicking Distance | 19.7 | 7.4 | 22 | 8.9 | 13 | 20 | 5.3 | 20.1 | 5.8 | 5 |
| Ramírez-Campillo et al. (80) 60s interset rest | Soccer | Kicking Distance | 18.9 | 5.0 | 21.8 | 4.1 | 13 | 20 | 5.3 | 20.1 | 5.8 | 5 |
| Ramírez-Campillo et al. (80) 120s interset rest | Soccer | Kicking Distance | 19.3 | 7.0 | 21.6 | 7.0 | 11 | 20 | 5.3 | 20.1 | 5.8 | 4 |
| Ramirez-Campillo et al. (92) fixed | Soccer | Kicking Distance | 40.8 | 5.6 | 41 | 6.4 | 25 | 39.8 | 5.8 | 40.2 | 5.5 | 12 |
| Ramirez-Campillo et al. (92) optimal | Soccer | Kicking Distance | 38.8 | 6.1 | 39.7 | 8.3 | 24 | 39.8 | 5.8 | 40.2 | 5.5 | 12 |
| Ramírez-Campillo et al. (94) | Soccer | Kicking Distance | 32.1 | 7.6 | 31.5 | 8.6 | 20 | 33.6 | 8.7 | 37.8 | 9.7 | 19 |
| Vera-Assaoka et al. (96) early | Soccer | Kicking Distance | 25.7 | 3.8 | 30.3 | 5.1 | 16 | 24.1 | 3.0 | 23.8 | 3.7 | 16 |
| Vera-Assaoka et al. (96) late | Soccer | Kicking Distance | 37.8 | 5.5 | 42 | 6.7 | 22 | 35.8 | 5.5 | 35.4 | 6.0 | 22 |
| Guadie (46) | Handball | Speed dribbling | 10.369 | 0.471 | 9.3109 | 0.682 | 11 | 10.51 | 0.521 | 10.2 | 0.696 | 11 |
| Sharma and Multani (77) | basketball | Speed dribbling | 26.95 | 4.32 | 30.6 | 2.47 | 20 | 27.3 | 3.85 | 27.05 | 3.41 | 20 |
| Çalışkan and Arikan (99) | Soccer | Speed dribbling | 30.48 | 3.16 | 27.1 | 1.89 | 13 | 30.09 | 3.22 | 27.07 | 1.82 | 12 |
| Saunders et al. (71) | Running | Stride rate | 84.3 | 2 | 84.4 | 1.4 | 7 | 86.5 | 4.7 | 86.4 | 4.7 | 8 |
| Giovanelli et al. (89) | Running | Stride rate | 2.92 | 0.16 | 2.98 | 0.18 | 13 | 2.9 | 0.14 | 2.91 | 0.12 | 12 |
| Ache-Dias et al. (90) | Running | Stride rate | 3.31 | 0.25 | 3.47 | 0.22 | 9 | 3.24 | 0.34 | 3.30 | 0.23 | 9 |
| Gomez-Molina et al. (93) | Running | Stride rate | 2.91 | 0.12 | 2.86 | 0.13 | 14 | 2.95 | 0.12 | 2.97 | 0.2 | 11 |

EG, experimental group; CG, control group, SD, standard deviation
